# Zika Virus Envelope Domain III Recombinant Protein Delivered With Saponin-Based Nanoadjuvant From *Quillaja brasiliensis* Enhances Anti-Zika Immune Responses, Including Neutralizing Antibodies and Splenocyte Proliferation

**DOI:** 10.3389/fimmu.2021.632714

**Published:** 2021-03-04

**Authors:** Samuel Cibulski, Ana Paula Muterle Varela, Thais Fumaco Teixeira, Martín Pablo Cancela, Patrícia Sesterheim, Diogo Onofre Souza, Paulo Michel Roehe, Fernando Silveira

**Affiliations:** ^1^Laboratório de Biotecnologia Celular e Molecular, Centro de Biotecnologia—CBiotec, Universidade Federal da Paraíba, João Pessoa, Brazil; ^2^Laboratório de Virologia, Departamento de Microbiologia Imunologia e Parasitologia, Universidade Federal do Rio Grande do Sul, Porto Alegre, Brazil; ^3^Laboratório de Genômica Estrutural e Funcional, Centro de Biotecnologia, Universidade Federal do Rio Grande do Sul, Porto Alegre, Brazil; ^4^Centro de Cardiologia Experimental, Instituto de Cardiologia/Fundação Universitária de Cardiologia, Porto Alegre, Brazil; ^5^Departamento de Bioquímica, Instituto de Ciências Básicas da Saúde, Universidade Federal do Rio Grande do Sul, Porto Alegre, Brazil; ^6^Departamento de Desarrollo Biotecnológico, Instituto de Higiene, Facultad de Medicina, Universidad de la República (UdelaR), Montevideo, Uruguay

**Keywords:** ISCOM, Zika virus, flavivirus, emerging diseases, Quillajaceae

## Abstract

Nanoadjuvants that combine immunostimulatory properties and delivery systems reportedly bestow major improvements on the efficacy of recombinant, protein-based vaccines. Among these, self-assembled micellar formulations named ISCOMs (immune stimulating complexes) show a great ability to trigger powerful immunological responses against infectious pathogens. Here, a nanoadjuvant preparation, based on saponins from *Quillaja brasiliensis*, was evaluated together with an experimental Zika virus (ZIKV) vaccine (IQB80-zEDIII) and compared to an equivalent vaccine with alum as the standard adjuvant. The preparations were administered to mice in two doses (on days zero and 14) and immune responses were evaluated on day 28 post-priming. Serum levels of anti-Zika virus IgG, IgG1, IgG2b, IgG2c, IgG3 were significantly increased by the nanoadjuvant vaccine, compared to the mice that received the alum-adjuvanted vaccine or the unadjuvanted vaccine. In addition, a robust production of neutralizing antibodies and *in vitro* splenocyte proliferative responses were observed in mice immunized with IQB80-zEDIII nanoformulated vaccine. Therefore, the IQB80-zEDIII recombinant preparation seems to be a suitable candidate vaccine for ZIKV. Overall, this study identified saponin-based delivery systems as an adequate adjuvant for recombinant ZIKV vaccines and has important implications for recombinant protein-based vaccine formulations against other flaviviruses and possibly enveloped viruses.

**Graphical Abstract:**
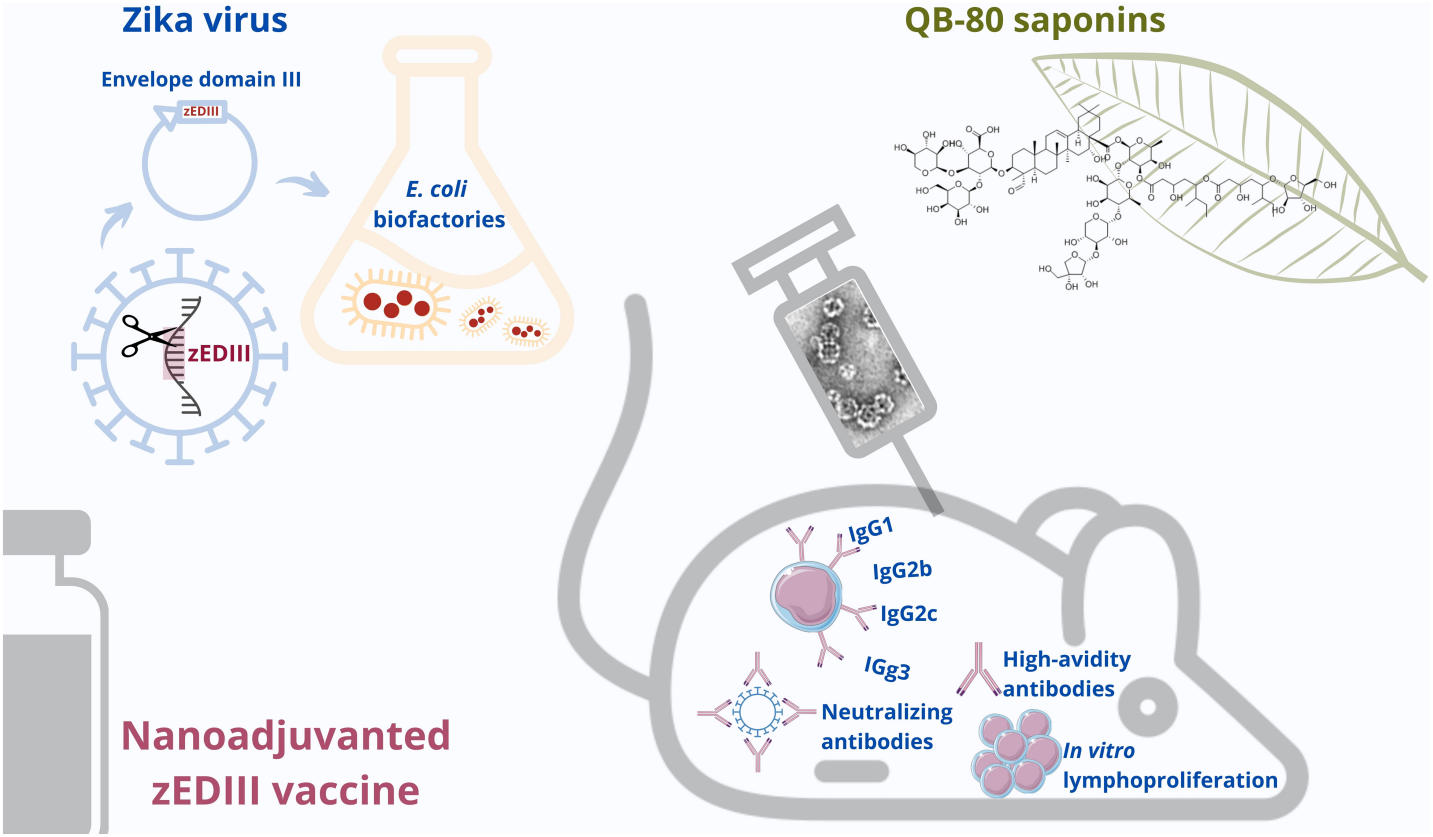
Saponin-based nanoadjuvanted ZIKV vaccine.

## Highlights

- zEDIII can be expressed as a soluble protein in *E. coli*.
- Subcutaneous administration of IQB80 zEDIII induces production of high titers of anti-ZIKV virus IgG containing neutralizing and high-affinity antibodies.
- zEDIII promotes *in vitro* splenocyte proliferation in IQB80 immunized mice.

## Introduction

Zika virus (ZIKV) is a recently re-emerged viral pathogen that is associated with severe neurological diseases, including Guillain-Barré Syndrome (GBS) and congenital Zika virus syndrome (CZS) (1). In order to prevent the consequences of such infections, a variety of ZIKV vaccines have been developed and evaluated in experimental animal models (2), though none has been licensed to date. Some groups of investigators have focused on the ZIKV E protein as an immunogen. ZIKV E is an envelope glycoprotein that is major protein responsible for the induction of protective immunity (3, 4). This is a transmembrane protein with three extracellular domains (DI, DII, and DIII), in which DIII has been shown to induce the most potent neutralizing activity against ZIKV, without inducing undesirable antibody-dependent enhancement (ADE) (4).

The use of recombinant protein antigens is reported as less reactogenic than whole inactivated pathogens; furthermore, recombinant proteins provide a number of advantages for production, compared to the inactivated pathogen, particularly in terms of safety and ease of scalability. However, recombinant-based vaccines usually require adjuvants. Adjuvants are added to vaccines either to stimulate innate immunity or to boost adaptive immune responses to antigens, ensuring efficient trafficking of effector and memory B and T cells (5, 6), thus improving cell-mediated immune responses (7–9).

Despite many decades of development, a very small number of adjuvants are currently approved for use in human vaccines (6). Currently, nanotechnology is being employed in response to the need for new adjuvant and delivery systems that can increase cellular and humoral immune responses. The use of nanoadjuvants in vaccine formulations allows enhanced immunogenicity and antigen stability, as well as targeted delivery and slow release (10). In this context, one group of adjuvants, the saponin-based adjuvants (SBA), has been proposed as an alternative to classical adjuvants for the design of new vaccines due to the ability of these compounds to trigger Th1 responses (9, 11–13). Recently, two vaccines that employ recombinant proteins as antigens were licensed for human use; one is a vaccine against herpes zoster (Shingrix™); another is against malaria (Mosquirix™). Both vaccine formulations contain QS-21, a saponin from *Quillaja saponaria*, as an adjuvant (8).

One critical issue concerning the use of saponins as vaccine adjuvants is their toxicity (14). For this reason, nanoadjuvants, such as immmunostimulating complexes or ISCOMs, have been formulated due to their reduced toxicities (8, 13, 15). Our research group previously reported that nanoadjuvants from *Q. brasiliensis* saponins are safe adjuvants in preclinical experiments (16). Furthermore, these nanoparticles are efficiently internalized by dendritic cells, promote high antibody titers, enhance delayed-type hypersensitivity (DTH) responses and stimulate the proliferation of Th1 lymphocyte subsets (17). Moreover, these nanoadjuvants, in the absence of antigen (ISCOM-matrices), generate a local and transient immunocompetent environment, with overexpression of cytokine and chemokine genes related to inflammation (18). Notably, the recombinant spike protein antigen, recently recovered from SARS-CoV-2, was formulated with a *Q. saponaria* saponin-based nanoadjuvant (Matrix-M). This putative vaccine is currently in phase 1/2 clinical trials as a possible candidate for fighting the SARS-CoV-2 pandemic (19, 20).

In this study, an experimental vaccine was prepared using a recombinant ZIKV E domain III (zEDIII) antigen. The zEDIII was obtained using an *Escherichia coli* and used as an immunogen in a nanoadjuvanted formulation (ISCOMs with the *Q. brasiliensis saponins*, IQB80). The nanoadjuvant-based vaccine formulation was tested in mice and its immunogenicity was evaluated in comparison to an equivalent vaccine preparation that was adjuvanted with alum, a standard adjuvant used in vaccines for humans.

## Materials and Methods

### Cells and Viruses

The ZIKV strain 17 (ZIKV 17), isolated from a patient in São Paulo city, Brazil (21), was propagated in Vero cells (CRL-1586, originally obtained from ATCC), and quantified by standard plaque-forming assays. Viral stocks were kept at −80°C until use. The cells were multiplied in Dulbecco's Modified Eagle Medium (DMEM, Gibco, USA) supplemented with 10% fetal bovine serum (Gibco, USA) and antibiotics (penicillin 100 U/mL; streptomycin 100 μg/mL) (Gibco, USA). Cells were grown at 37°C in an atmosphere of 5% CO_2_ and were used for virus multiplication and neutralization tests.

### Cloning and Recombinant Expression of ZIKV EDIII in *Escherichia coli*

Viral RNA from Vero cells infected with ZIKV 17 was isolated with TRIzol (Invitrogen, USA), according to the manufacturer's instructions. Total RNA was reverse transcribed using GoScript™ Reverse Transcriptase (Promega, USA). The coding sequence of the zEDIII domain (306 bp, amino acid residues 302–403) of the E protein was amplified by PCR using ZIKV 17 cDNA and the specific primers ZIKV_EDIII_F (5′-GTGTCATACTCCTTGTGTACT-3′) and ZIKV_EDIII_R (5′-TTAACTCCTGTGCCAGTGGTG-3′). The underlined sequence corresponds to a stop codon inserted to stop translation. The ZIKV 17 genome sequence is available under GenBank accession number MH882542. The fragment obtained with these primers was ligated into the Champion™ pET-SUMO TA expression vector in frame with the coding sequence of the SUMO protein (Thermo Fischer Scientific, USA). The recombinant pET-SUMO-zEDIII plasmid was extracted and its sequence confirmed by Sanger DNA sequencing.

To produce the recombinant zEDIII protein, pET-SUMO-zEDIII was transformed into *E. coli* strain BL21 (DE3) (Thermo Fisher Scientific) following the usual methodology. Bacterial cells were grown in LB media with 50 μg/mL kanamycin with shaking (200 rpm) at 37°C. When OD_600_ nm reached 0.8, IPTG was added to a final concentration of 0.1 mM and recombinant protein synthesis was induced for 3 h at 20°C. Cells were harvested by centrifugation and resuspended in cell lysis buffer (50 mM Tris pH 8.0, 500 mM NaCl, 10% glycerol, and 10 mM imidazole). Three cycles of freeze (-80°C)/thaw (42°C) were performed and the sheared cell suspension was sonicated with five pulses of ultrasound (30 s each, potency 60%) with an ultrasonic homogenizer 4710 (Cole-Palmer Instruments, USA). After centrifugation at 15,000 × g for 60 min at 4°C, the soluble fraction containing recombinant zEDIII protein was purified by Ni Sepharose High Performance histidine-tagged protein purification resin according to the manufacturer's instructions (GE Healthcare, USA). Elution of the zEDIII recombinant protein was performed using a discontinuous gradient of 200, 500, and 1,000 mM imidazole in lysis buffer. Eluted fractions were dialyzed against 2 L of dialysis buffer (50 mM Tris-HCl pH 8.0, 0.5 M NaCl).The protein concentration was determined using Qubit protein assay kit (ThermoFisher Scientific, USA) and protein purity was assessed by SDS-PAGE and further confirmed by Western blot with anti-ZIKV 17 hyperimmune murine sera. In order to remove endotoxins, the purified protein was chromatographed in a polymyxin B-agarose column (Sigma-Aldrich, USA).

### Immunogenicity and Preparation of Recombinant zEDIII Polyclonal Antisera

To assess the immunogenicity of recombinant zEDIII immunogenicity, 10 μg of purified protein were mixed with 10 μg of QB-80 saponins (total volume of 100 μL). Six C57BL/6N mice (6 weeks old at the first immunization) were inoculated subcutaneously (s.c.) at days 0 and 14. Sera were collected at day 30 and day 60 following the first immunization by retro-orbital bleeding. zEDIII protein-specific IgG antibody titers in individual murine sera were measured by ELISA (as described below).

### Indirect Immunofluorescence Microscopy

In order to determine whether sera from mice immunized with zEDIII would react with viral E protein in ZIKV-infected Vero cells, an indirect immunofluorescence (IFI) assay was performed. Briefly, ZIKV-infected Vero cells (m.o.i. 0.1) were fixed 72 h post-infection in 4% paraformaldehyde for 15 min at 4°C. Cells were then washed with PBS and permeabilized with 0.05% of Triton X-100 in PBS for 30 min. A pool of sera from mice immunized with zEDIII was used as primary antibody (at an appropriate dilution in PBS). Cells were then incubated at 37°C for 1 h, washed three times with PBS and incubated with Alexa Fluor™ 488 goat/anti-mouse conjugate (Thermo Fisher Scientific, USA). After 30 min of incubation at room temperature, cells were washed and stained with DAPI and phalloidin conjugated with Alexa Fluor™ 568 (Thermo Scientific). Cells were observed in the EVOS™ FL Auto Imaging System (Invitrogen, USA). Images were obtained using camera-specific software and processed using ImageJ.

### Nanoadjuvant Vaccine Preparation

*Q. brasiliensis* (A. St.-Hil. Et Tul) Mart. leaves were collected in Canguçu, RS, Brazil (31°23′42″ S-52°40′32″ W) (voucher ICN 142953, deposited at the Herbarium of the Federal University of Rio Grande do Sul). The extraction and purification of saponins were carried out as previously described (22).

The nanoadjuvanted formulation with the zEDIII antigen was prepared by the modified ethanol injection technique (17, 23). Briefly, the zEDIII protein solution (1 mg/mL or 0.2 mg/mL) was added to a mixture of *Q. brasiliensis* saponins fraction (QB-80, 1 mg/mL). Ethanol-dissolved cholesterol (Sigma-Aldrich, USA) and di-palmitoylphosphatidyl-choline (Avanti Polar Lipids, USA) were immediately injected into the mixture, which was then stirred for 48 h at 4°C.

This procedure resulted in the nanoadjuvant derived from QB-80, which was named IQB80. In order to check the toxicity of the nanoadjuvant, hemolysis tests were performed, as previously described (17, 18, 24), and none of the concentrations tested (ranging from 100 to 10 μg) have been shown to cause membrane damage (data not shown). The experimental vaccine formulations were prepared under aseptic conditions, filtered through 0.22 μm filters and kept at 4°C until use.

### Mice Immunization Protocol

Female C57BL/6N (5–6-weeks old) mice were purchased from the Experimental Cardiology Center of Institute of Cardiology of Rio Grande do Sul (CCE/IC-FUC) and acclimatized for 72 h prior to use. Mice were maintained under controlled temperature; food and tap water were provided *ad libitum*. All procedures were carried out in accordance with the National Council for Animal Experimentation Control and approved by the institutional Animal Care and Use Committee (CEUA) under protocol UP 5402/17.

Fifty animals (*n* = 10 per group) were inoculated subcutaneously (the scruff of the neck) twice, on days 1 and 14, with 10 μg of zEDIII protein antigen (no adjuvant group), or with either Alhydrogel® adjuvant (100 μg, InvivoGen, USA), 10 μg IQB80 zEDIII (10 μg of saponin with 10 μg of zEDIII) or 2 μg IQB80 zEDIII (10 μg of saponin with 2 μg of zEDIII). In addition, saline-treated animals were included as a control group. The concentration of IQB80 (10 μg) used in this study is defined as the saponin concentration within the particles. Mice were bled prior to inoculations (on days 0 and 14) and 2 weeks after the second immunization (day 28). Sera were frozen until processed. On day 28, three mice from each group were euthanized, and the spleens aseptically removed for *in vitro* splenocyte cultures. In accordance with the 3Rs for animal welfare (Replacement, Reduction and Refinement), we did not include a mice group inoculated with IQB-80, as we have background that QB nanoadjuvants did not show any significant interference (unpublished studies).

### Immunoassays for Antibodies

#### Determination of zEDIII-Specific Antibodies by Enzyme Linked Immunosorbent Assay (zEDIII-ELISA)

Anti-zEDIII IgM, IgG, and anti-isotypes IgG1, IgG2b, IgG2c, and IgG3 (all of these goat anti-mouse horseradish peroxidase conjugates; Southern Biotech, USA) were determined by ELISA. Briefly, ELISA plates (Greiner Bio-One, Germany) were coated with zEDIII protein (2 μg/mL) in phosphate buffered saline (PBS, 100 μL/well, pH 7.0). The anti-zEDIII ELISA protocols were essentially performed as previously described (17). As substrate, o-phenylenediamine dihydrochloride (OPD, Amresco, USA) were used. Optical densities (OD) were measured in an ELISA plate reader (SpectraMax, Molecular Devices, USA) at 492 nm. A pool of positive sera was used to construct a standard curve. Antibody titers were expressed in arbitrary units per mL (AU/mL).

#### zEDIII IgG Avidity

Anti-zEDIII IgG avidity was determined on mice sera by 5M urea denaturation, as described elsewhere (25). Briefly, sera appropriately diluted in PBS-TWEEN® 20 (PBS-T20), were added in quadruplicate to zEDIII-coated ELISA plates (as described above). After 1h of incubation at 37°C, plates were washed three times with PBS-T20 and 100 μL of 5M urea were added to half of the wells. Wells that did not receive urea were filled with PBS-T20. Plates were incubated for 30 min at 37°C and washed three times with PBS-T20. Subsequently, plates were incubated with anti-mouse IgG peroxidase conjugate (Sigma-Aldrich) for 1h at 37°C. Subsequent procedures, stopping and readings were carried out as for the ELISAs above. IgG avidity was expressed as an avidity index (AI), calculated as follows: AI = (mean Abs of urea-treated wells/mean OD urea-untreated wells) × 100%.

#### Determination of Anti-ZIKV Neutralizing Antibodies by Plaque Reduction Neutralization Test (PRNT)

The serum neutralizing antibody titers were determined by plaque reduction neutralization tests (PRNT) in Vero cells. Sera from individual animals, collected on day 28 post priming, were heat inactivated by incubation at 56°C for 30 min. Serum from animals in each group were pooled and, then tested at three dilutions in DMEM (1:5, 1:10, and 1:25), mixed with an equal volume of 50 PFU (plaque forming units)/well of the ZIKV 17, followed by incubation at 37°C for 1 h. The serum-virus mixture was added in duplicate to confluent Vero cells and incubated at 37°C for 1 h. The cells were plated 24 h beforehand in a 24-well plate (Greiner Bio-One, Germany), using a seeding density of 3 × 10^5^ cells/well in a 1 mL volume. Next, the medium was replaced with 1 mL of DMEM containing 1% low melting point agarose. After 96 h, the plates were fixed using 10% formalin solution (1 mL/well) and stained with crystal violet (1% in PBS, w/vol) for plaque counting. The experiments were performed twice in each batch of tests.

### Splenocyte Proliferation Assay

Fourteen days after the booster administration, spleens were collected under aseptic conditions, immersed in RPMI 1640 medium (Invitrogen, USA) and mechanically dissociated to obtain a homogeneous cell suspension as described elsewhere (17, 26). Either zEDIII antigen (10 μg/mL), inactivated ZIKV strain 17 (10 μg/mL) or medium with an unrelated antigen (inactivated bovine herpesvirus, 10 μg/mL) was used for *in vitro* re-stimulation antigen.

For antigen production, the supernatant medium of ZIKV-infected cells was clarified using low speed centrifugation and filtered through a 0.45 μm filter. Viral particles were purified by ultracentrifugation at 100,000 × *g* on a 20% sucrose cushion. The viral pellet was inactivated with 0.05% formalin at 22°C for 7 days. The formalin was then removed by extensive dialysis against PBS (pH 7.2). Antigen concentration was then carried out using Amicon® Ultra Centrifuge Filters (molecular weight cut-off of 30 K, Sigma-Aldrich, USA). Antigen inactivation was confirmed by three passages in Vero cells. The amount of viral protein antigen was quantified by fluorimetry in a Qubit apparatus (Thermo Fisher Scientific, USA) using the Qubit protein assay kit.

### Statistical Analyses

The significance of differences was assessed using the nonparametric Kruskal-Wallis test with Dunn's posttest. GraphPad Prism version 8 (GraphPad Software, USA) was used for data analysis. Results are expressed as the mean or median value from individuals in each group ± SEM. A *p* ≤ 0.05 was considered statistically significant.

## Results and Discussion

### Expression, Purification, and Characterization of zEDIII

**Figure 1:**
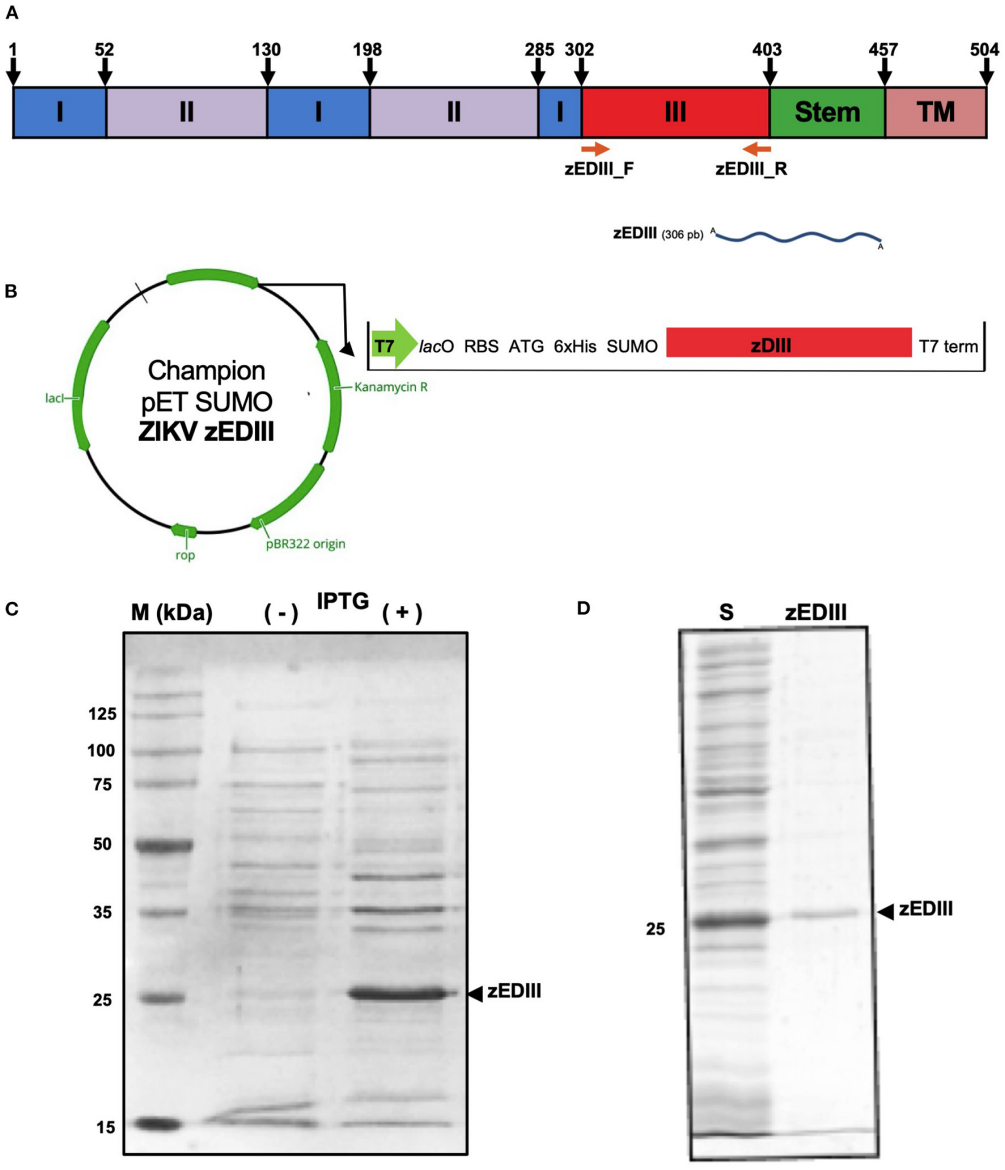
Expression of zEDIII in *E. coli*. **(A)** Schematic diagram of ZIKV envelope protein. Domain III is marked in red, and this region was used as a template for RT-PCR. **(B)** zEDIII region cloned in the Champion pET SUMO. **(C)** Total cellular proteins of the *E. coli* BL21 (DE3) harboring the recombinant plasmid analyzed by SDS-PAGE under reducing conditions, followed by Coomassie blue staining. Lane 1, molecular weight marker (M); Lane 2, total protein sample from non-induced *E. coli*, used as a negative control; Lanes 3, total protein from IPTG induced *E. coli* collected 6 h post IPTG induction (1 mM). The expression of the recombinant zEDIII protein is indicated by an arrowhead. **(D)** SDS-PAGE of solubilized zEDIII in lysis buffer (S) and after Ni-chelating chromatography (zEDIII).

In this study, the coding sequence of zEDIII (Figure 1A) was amplified from an autochthonous ZIKV (strain 17) and cloned into the expression vector, Champion™ pET SUMO (Figure 1B). Using this system, the expression of a recombinant protein with the expected molecular weight (His6-SUMO-zEDIII; ~24 KDa) was easily visualized by SDS-PAGE (Figure 1C). The zEDIII was detected 1 h after IPTG induction. The highest levels of accumulation of this protein were achieved after 6 h of incubation. However, a major challenge associated with recombinant protein production in *E. coli* is the generation of large quantities of soluble, functional protein (27). Further analysis with SDS-PAGE indicated that zEDIII was retained in inclusion bodies (data not shown). In order to enhance solubility of the zEDIII recombinant protein, the BL21 (DE3) were induced with IPTG 0.1 mM and incubated at 20°C for 3 h. Using this approach, ≥70% of the recombinant zDEIII protein was found in a soluble form and could easily be purified using Ni-sepharose resin. The recombinant protein was efficiently isolated from *E. coli* host proteins to >95% of purity (analyzed by SDS-PAGE) (Figure 1D and Supplementary Figure 1).

### Recombinant ZIKV EDIII Antigen Is Immunogenic in Mice and zEDIII Antisera React With Viral E Protein in ZIKV-Infected Vero Cells

**Figure 2:**
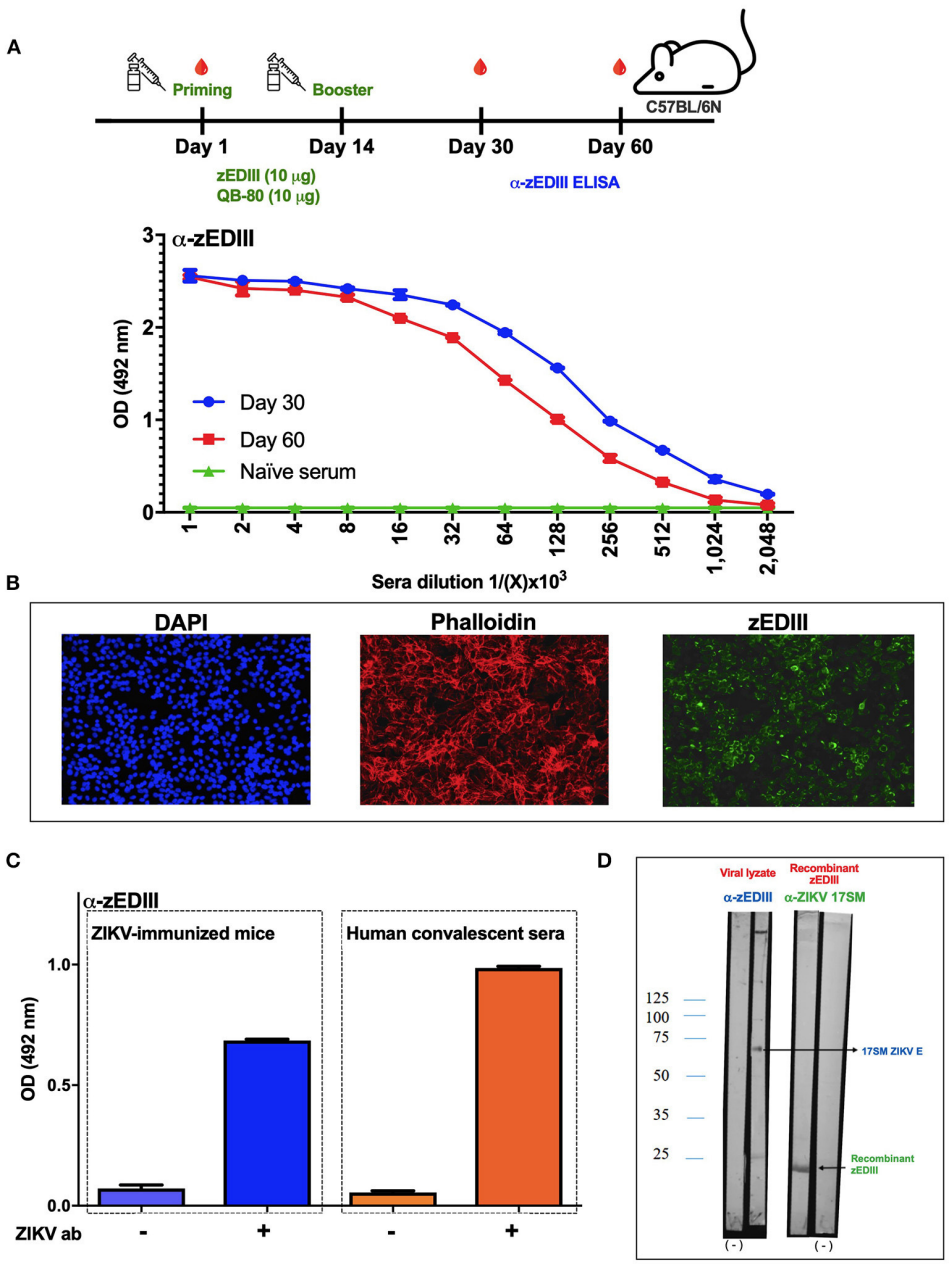
Recombinant ZIKV EDIII antigen is immunogenic in mice and recognizes native ZIKV E protein. **(A)** Vaccination schedule. The syringe drawings indicate the priming and booster immunizations with 10 μg recombinant zEDIII plus 10 μg QB-80 saponins. Drops at days 30 and 60 indicate blood draws. Below, naïve, and 30- and 60-days post-priming sera were titrated by doubling dilutions and tested against zEDIII recombinant antigen in ELISA. Individual titers were presented as mean ± S.E.M. **(B)** Antibodies against recombinant zEDIII recognize native protein in ZIKV-infected Vero cells. Mouse sera raised against recombinant zEDIII were used for immune fluorescence assays. **(C)** Sera from mice vaccinated with an inactivated ZIKV 17 antigen and sera from humans infected with ZIKV recognized the recombinant zEDIII in ELISA. **(D)** Western blot analysis showed that sera from mice immunized with a recombinant zEDIII (α-zEDIII) recognized the ZIKV E protein (ZIKV 17 E) and that sera from mice vaccinated with an inactivated ZIKV 17 antigen (α-ZIKV 17) recognized the recombinant zEDIII. (–) represents the background control (lanes incubated with a pool of pre-immune sera).

The immunogenicity of the purified zEDIII protein was initially tested by immunizing mice with two doses of zEDIII (10 μg per dose) plus 10 μg of QB-80 saponins. After the immunization schedule, IgG titers were >2,048,000 (Figure 2A). The mice were followed until day 60 post-priming when, at that date, the average antibody titers reached up to 1,024,000 (Figure 2A). These results suggest that two doses of the zEDIII protein (10 μg) with QB-80 saponins were sufficient to elicit strong humoral responses, as demonstrated by the high levels of ELISA-reactive anti-zEDIII antibodies.

Furthermore, tests were conducted to determine whether sera from mice immunized with zEDIII would react with the native E protein in ZIKV-infected Vero cells. For this, Vero cells infected with ZIKV 17 were submitted to indirect immunofluorescence assays (IFA) using a pool of sera from mice immunized with zEDIII as primary antibody (collected from mice used in the immunogenicity study described above). IFA results showed that the sera from mice immunized with zEDIII did react with viral E protein in ZIKV-infected Vero cells (Figure 2B). As expected, ZIKV-specific antibodies were not detected in sera from naïve mice (Supplementary Figure 2).

Additionally, to confirm the authenticity of the purified protein, we examined its binding potential to a mouse polyclonal serum generated against ZIKV 17 (Figure 2C). Furthermore, purified zEDIII was also assayed against human sera from a confirmed case of ZIKV infection. For these, ELISA plates coated with 2 μg/mL of zEDIII were used to detect anti-ZIKV antibodies collected from ZIKV 17-immunized mice or human convalescent serum. The results (summarized in Figure 2C) showed that sera from ZIKV 17-immunized mice, as well as human convalescent serum, were capable of binding to the recombinant zEDIII. This human serum sample was recovered from a collaborator which had a confirmed case of ZIKV infection in Rio de Janeiro city, Brazil, and the ZIKV binding capacity was confirmed by immunoperoxidase monolayer assay (IPMA) against ZIKV 17-infected Vero cells (Supplementary Figure 3), in addition to ELISA (as described in this study). The recognition of the ZIKV 17 E protein by serum from recombinant zEDIII-immunized animals was also confirmed by Western blot (Figure 2D). Likewise, the recognition of the zEDIII recombinant protein by ZIKV 17- immunized mice sera was demonstrated (Figure 2D).

### Immunization Using zEDIII and *Q. brasiliensis* Saponin-Based Nanoparticulate Adjuvant Elicited Specific Immune Responses in Mice Against Zika Virus

**Figure 3:**
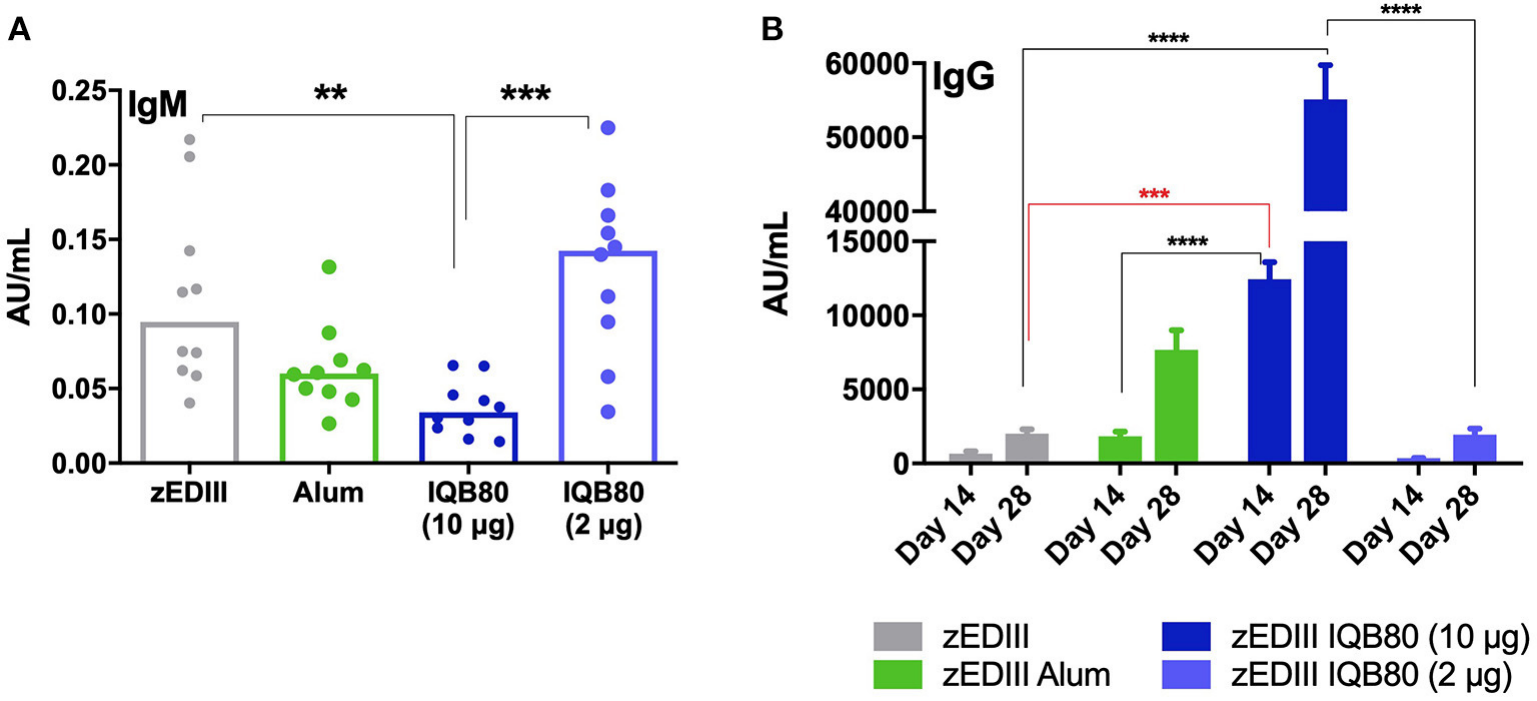
Antibody response in immunized mice. **(A)** Serum titers of anti-zEDIII IgM at 2 weeks post-priming (day 14). **(B)** Total anti-zEDIII IgG at day 14 and 28 post second vaccination. The median value is indicated by a line, and dots indicate individual values. Statistical analyses were performed using a non-parametric Kruskal-Wallis test with Dunn's posttest to compare each group with the control group (non-adjuvant zEDIII). Additionally, a multi comparison test was used to compare the means of all groups (again using the Kruskal-Wallis test with Dunn's posttest). Significant differences are indicated: **(*p* ≤ 0.01), ***(*p* ≤ 0.001), ****(*p* ≤ 0.0001).

The presence of specific anti-zEDIII antibodies was evaluated by ELISA 2 weeks after the first (priming) and second (booster) immunizations. On day 14, significant differences were obtained to increase IgM antibodies among groups immunized with different vaccine formulations (Figure 3A). It is striking that the levels of IgM antibodies induced by IQB80 (10 μg) were significantly lower than those induced by the other groups, including the non-adjuvanted zEDIII group (*p* = 0.0030) and IQB80 (2 μg) (*p* = 0.0007) (Figure 3A).

With regard to IgG antibodies, a significant increase in total anti-zEDIII IgG antibodies was observed in the group of mice immunized with nanoadjuvanted antigen (as IQB80, 10 μg), when compared to the group immunized with antigen only (*p* = 0.0002). Interestingly, mice from the IQB80 (10 μg) group showed a large amount of anti-zEDIII IgG with only one dose of the vaccine (mean 12,453 AU). This was higher than that detected in animals vaccinated with two doses of alum-adjuvanted zEDIII (mean 7,677 AU) (Figure 3B). No significant increase in specific anti-zEDIII IgG was observed in the Alum and IQB80 (2 μg) groups.

As expected, the immune response in all groups was boosted after the second dose of vaccine (Figure 3B). In particular, both groups that were immunized with SBA vaccines, showed significant differences with respect to the antigen-only control group. All serum samples collected on day zero (pre-immunization) were negative for the presence of anti-zEDIII IgG (data not shown).

The significantly low IgM and high IgG titers, particularly in the IQB80 (10 μg) immunized animals at day 14, could be explained by a rapid switch from IgM to IgG. Although ISCOMs induce strong and persistent T cell responses (13, 28), such complexes are particularly acknowledged for their powerful adjuvant effect on the stimulation of B-cell responses and antibody production. In particular, immunization with SBA promotes high-affinity antibodies (25) (as noted and further discussed in this work) and enhanced frequencies of antigen-specific memory B cells (13), resulting in higher frequencies of responding memory B cells and higher titers of protective neutralizing antibodies. Therefore, we believe that the immunization of zEDIII with IQB80 shapes germinal centers (GC), where the expansion, selection, and affinity maturation of zEDIII-specific B cells takes place during primary immune responses (29).

**Figure 4:**
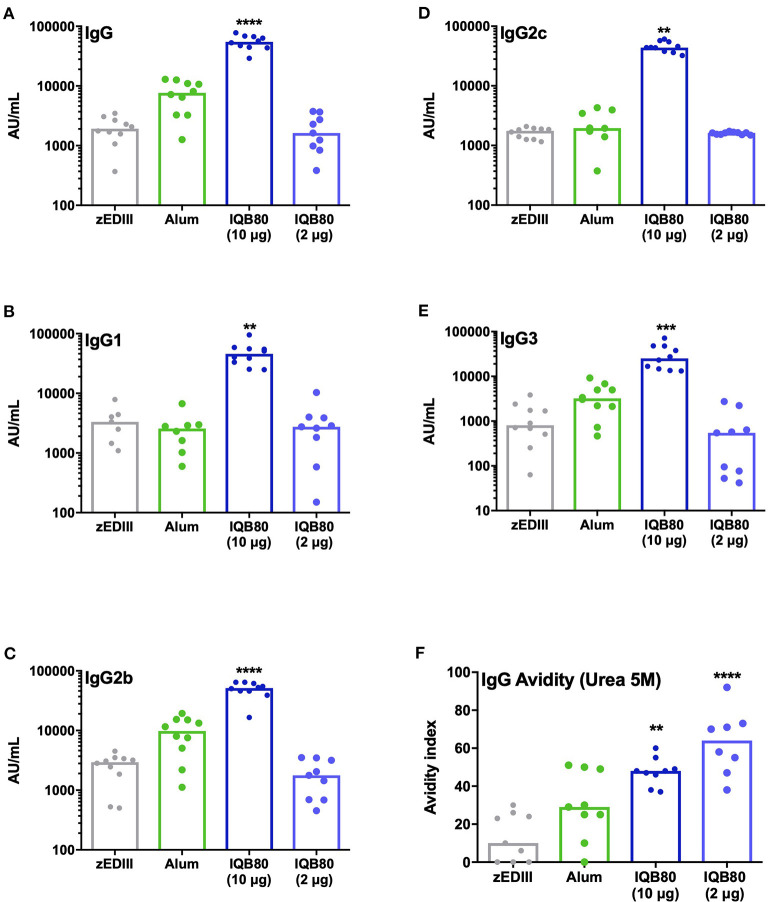
IQB80 enhances specific anti-zEDIII immune responses. Antibody titers of **(A)** anti-zEDIII IgG, **(B)** IgG1, **(C)** IgG2b, **(D)** IgG2c, and **(E)** IgG3 were determined 2 weeks after the second immunization (day 28). **(F)** Avidity index of anti-zEDIII IgG antibodies measured at day 28. Median values are indicated by lines and dots indicate individual values. Statistical analyses were performed in all cases **(A–F)** using the non-parametric Kruskal-Wallis test with Dunn's posttest to compare each group with the control group (zEDIII antigen alone). Significant differences are indicated: **(*p* ≤ 0.01), ***(*p* ≤ 0.001), ****(*p* ≤ 0.0001).

Figure 4A shows the levels of anti-zEDIII IgG at day 28 post priming. A highly significant increase in anti-zEDIII IgG was verified in mice immunized with the IQB80 (10 μg) when compared with mice immunized with zEDIII without adjuvant (*p* < 0.0001). This increase was >27-fold. The group of mice immunized with the alum adjuvanted-zEDIII preparation also presented an increase in levels of anti-zEDIII IgG; however, this was not statistically significant (*p* = 0.0762). The increase in IgG levels in mice that received the alum-adjuvanted vaccine was 3.8-fold superior in relation to mice that received antigen only. Interestingly, the levels of antibodies detected in mice immunized with antigen only did not differ significantly from the group receiving 5 times less antigen in the nanoadjuvant formulation (IQB80 2 μg) (*p* > 0.9999), while still being able to trigger humoral immune responses.

The results shown in Figures 4B–E illustrate the serum levels of zEDIII-specific IgG1, IgG2b, IgG2c, and IgG3 determined 2 weeks after the second immunization with all preparations tested. In mice vaccinated with IQB80 (10 μg), significant increases in IgG1 (*p* = 0.0071), IgG2b (*p* < 0.0001), IgG2c (*p* = 0.0002), and IgG3 (*p* = 0.0001) were assessed, when compared to the non-adjuvanted group. In addition, these responses are superior to those generated in mice inoculated with the alum-adjuvanted vaccine or IQB80 (2 μg). In mice, Th1 responses are usually associated with enhanced isotype switching to IgG2a/2c and IgG3 (which are promoted by INF-γ), whereas Th2 responses stimulate the production of IgG1 (which is promoted by IL-4) (30). In this context, and although not conclusive, the isotype pattern elicited by IQB80 indicates that it is capable of inducing an antibody response with a Th1-type bias, as evidenced by the high levels of IgG2c and the production of IgG3. In addition, the elevated titers of IgG1 suggest that Th2 CD4^+^ T cells are also involved in the response against the administered antigen.

The IgG isotype antibody titers in the alum group were similar to those found in mice vaccinated with a non-adjuvanted antigen. This suggests that the immunization regime applied in these groups did not significantly increase the zEDIII-specific antibody response. Furthermore, findings highlight differences in the isotype profile of mice immunized with alum or the IQB80 formulations. The pattern of the zEDIII-specific IgG response with alum as adjuvant is almost identical to that found with zEDIII alone. Finally, the nanoadjuvant IQB80 containing 2 μg of zEDIII was found to have a similar effect in this experimental vaccine (antigen sparing is regarded as crucial for epidemic/pandemic vaccine development). Any IgG isotypes analyzed were statistically different between the non-adjuvanted group and the group that received 5 times less antigen (in nanoadjuvant form).

Antibodies link the adaptive immune system with the effector mechanisms of the innate immune system. They form a bridge by combining antigen-binding sites with binding sites for many innate receptors and adaptor molecules. The effector mechanisms that will be triggered vary between the different immunoglobulin subclasses (31). Viral vaccine effectiveness studies have generally focused on the induction of antibodies that mediate neutralization, a proven correlate of protection. In addition to neutralization, viral-specific antibodies may mediate a number of Fc-receptor dependent functions, including complement-mediated lysis (mostly IgG2a/2c and IgG2b), phagocytosis (IgG2a/2c and IgG2b), and ADCC (especially IgG2a/2c, IgG2b, and IgG3). Indeed, the Fc-receptor binding activity of antibodies has been shown to increase the protective efficacy of broadly neutralizing antibodies and has been associated with protection against viral infections (32).

The avidity of IgG antibodies, elicited by vaccine formulations, was assessed on day 28 post-priming. The groups immunized with IQB80 nanoadjuvant (containing 10 μg or particularly 2 μg of zEDIII protein) demonstrated higher IgG-avidity, when compared to mice vaccinated with the non-adjuvanted formulation (zEDIII antigen only), and notably, when compared with the alum-adjuvanted formulation (Figure 4F). It is essential for adjuvants to generate antibodies with high avidity, in addition to generating a high titer of antibodies (33). High-avidity antibodies exhibited to be important role in the protection conferred by several viral, bacterial and malaria vaccines (34–39). This may correlate with a successful immune response, allowing an adequate protection of the vaccinated individual, and the rapid clearance of a potential infection.

Although antibody avidity is infrequently included as a measure of adjuvant efficacy when evaluating new compounds as adjuvants, this assessment adds valuable information that contributes to the understanding of mechanisms by which these components stimulate the immune system (40). Herein, we showed that antibodies elicited by the studied IQB80 formulations have a higher avidity than the antibodies elicited by alum formulations. Based on this, we propose that the humoral immune response elicited by *Q. brasiliensis* nanoadjuvants may trigger a more adequate immune response to combat ZIKV infections. High-avidity antibodies might improve immune protection, through improved recognition of virus particles, even at low viral loads, and trigger effector functions as neutralization, opsonization and complement activation. Thus, vaccine formulations with *Q. brasiliensis* nanoadjuvants may improve vaccine efficacy providing more robust protection as well as the potential for cross-protection, particularly important for vaccines against highly variable pathogens, such as RNA viruses (40, 41).

One critical issue concerning the use of saponins as vaccine adjuvants is its potential toxicity. As stated previously, IQB80 formulations showed no hemolysis *in vitro* and regarding *in vivo* toxicity, no signs of local toxicity (local swelling, loss of hair, and piloerection) was detected after subcutaneous administration of the two vaccine doses. This issue had already been addressed previously by our group, demonstrating the reduced toxicity of *Q. brasiliensis* saponins when compared to *Q. saponaria* saponins (especially Quil A®, a commercial saponin fraction of *Q. saponaria*). This was demonstrated using several *in vitro* assays (hemolytic activities, cell viability, brine shrimp lethality) as well as *in vitro* acute toxicity tests, that *Q. brasiliensis* saponins (QB-90 or QB-80) (17, 25, 26, 42). In addition to reduced toxicity, when these compounds were assembled as micellar formulations (ISCOM or ISCOM matrices), the hemolytic, cytotoxic and brine shrimp lethal activities were abolished (17, 18). As such, the saponins of *Q. brasiliensis* appear to be appealing alternatives to vaccines that may require a strong stimulation of the immune system.

### The zEDIII Vaccine, When Adjuvanted With *Q. brasiliensis* Saponin-Based Nanoadjuvant, Induces Neutralizing Antibodies, and Promotes Lymphoproliferative Responses

**Figure 5:**
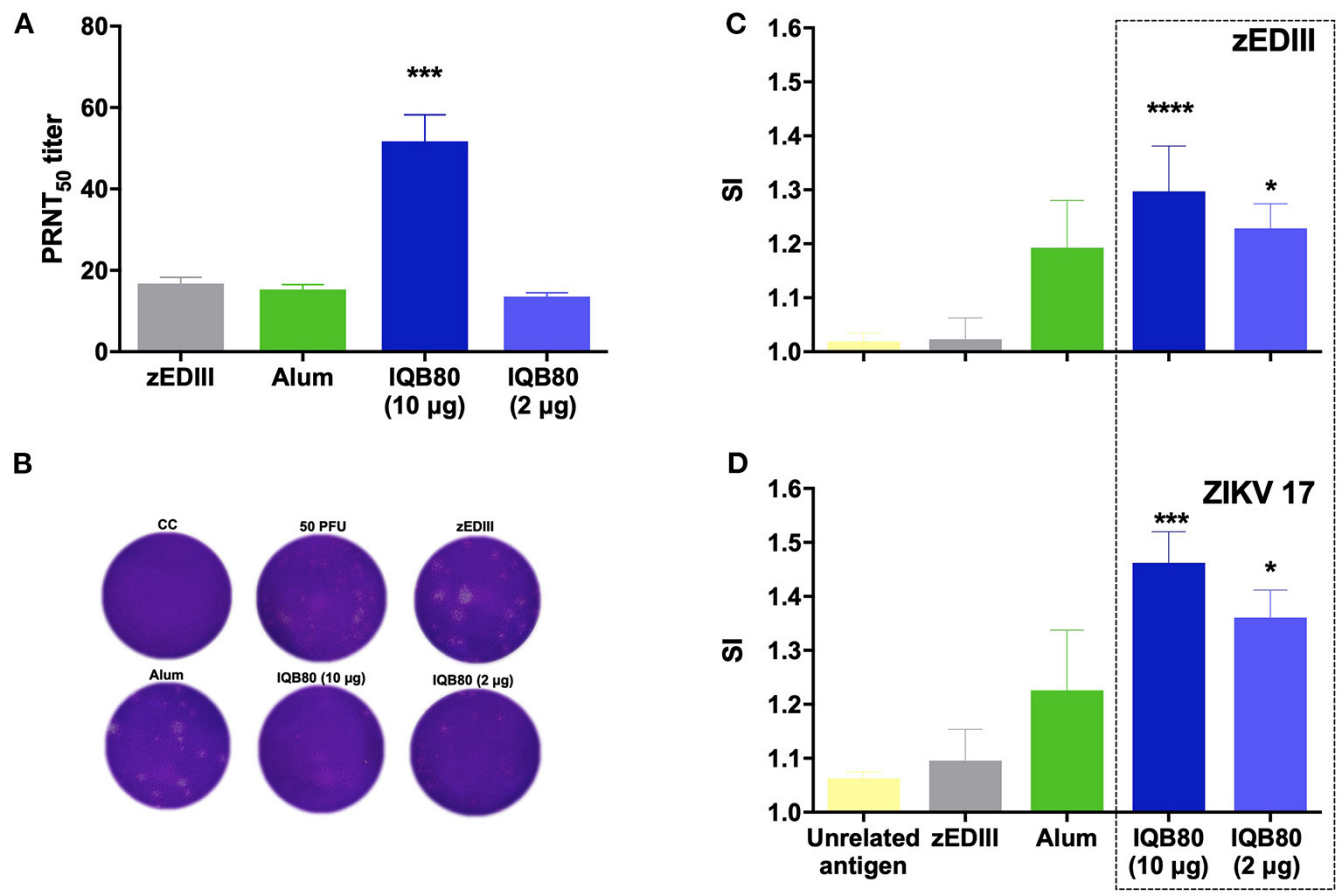
Plaque reduction neutralization assays (PRNT) and splenocyte proliferation responses in zEDIII-immunized mice. **(A)** Neutralizing antibody titers against ZIKV. Neutralization was determined by a standard plaque reduction neutralization test. Sera were diluted and incubated with 50 PFU of ZIKV 17, prior to the infection of Vero cells. PRNT_50_ titers is presented. **(B)** Crystal violet stained PRNT plates. **(C,D)** Splenocyte proliferation assays. Cell-proliferative responses (represented as stimulation index, SI) were measured 14 days after the last immunization. Splenocytes from each mouse were prepared and pulsed *in vitro* with purified recombinant zEDIII (10 μg/mL) **(C)** or an inactivated ZIKV 17 (10 μg/mL) **(D)**. Data are shown as mean ± SEM. Statistical analyses were performed using non-parametric Kruskal-Wallis test with Dunn's post test to compare each group with the control group (zEDIII without adjuvant). Significant differences are indicated: *(*p* ≤ 0.05), ***(*p* ≤ 0.001), ****(*p* ≤ 0.0001).

The levels of neutralizing antibodies induced by the zEDIII vaccine when formulated with the different adjuvants were evaluated by PRNT against the ZIKV strain 17 on day 28 after the first immunization. The nanoadjuvanted preparation with 10 μg of zEDIII induced a stronger neutralizing antibody response (Figures 5A,B). This response was significantly higher than that induced by the zEDIII antigen alone (*p* = 0.0004) or by alum (*p* = 0.0003) (Figure 5A). The neutralizing responses in the alum or IQB80 (2 μg) groups did not significantly differ from the responses generated by zEDIII alone (*p* = 0.9899). In sera from animals immunized with saline, no neutralizing antibody responses were identified (data not shown).

There is a considerable history of successful vaccines against flaviviruses (43). Japanese encephalitis virus (JEV), Tick-borne encephalitis virus (TBEV), and Yellow fever virus (YFV) vaccines have correlates of protection (44). In YFV and JEV vaccines, neutralizing antibodies demonstrated to be important protective markers, while total antibody responses and neutralizing antibodies are correlates for the TBEV protection (45–47). The experience with these classical flavivirus vaccines joined with data currently available for ZIKV experimental vaccines propose that neutralizing antibodies are expected to be important in protecting against ZIKV associated-diseases (2, 48, 49). Thus, the recombinant E antigen, especially the nanoadjuvanted preparation with 10 μg of zEDIII, which generates a considerable amount of neutralizing antibodies, can be a promising vaccine platform for ZIKV.

In order to investigate the effects of the immunogens on cellular immune responses, *in vitro* cell proliferation assays were performed in mice immunized with the different vaccine formulations and exposed to either recombinant zEDIII (10 μg/mL) or to inactivated ZIKV 17 virus (10 μg/mL). Significant proliferation was observed in mice immunized with both IQB80 preparations (IQB80 10 and 2 μg) (Figures 5C,D). The highest responses in the proliferation assays were recorded in the group that was vaccinated with the nanoadjuvanted IQB80 preparation containing 10 μg of zEDIII, following stimulation with either zEDIII antigen (*p* < 0.0001) or inactivated ZIKV antigen (*p* = 0.0005). The proliferation rates of the group that was vaccinated with zEDIII without adjuvant did not differ significantly from mice immunized with saline and pulsed with an irrelevant antigen (*p* > 0.05).

In addition to eliciting an improved response to antigens, an adjuvant should ideally reduce the amount of antigen needed to induce immune stimulation. This benefit, known as antigen sparing, is particularly sought during pandemic outbreaks as it enables the production of larger numbers of vaccine doses (25, 50–52). With regard to the nanoadjuvant preparation containing 2 μg of zEDIII, we noted a remarkable dose-sparing effect that allowed the formulation of preparations with a five-fold decrease in the amount of antigen/dose per dose (from 10 to 2 μg/dose) while still triggering neutralizing responses at the same magnitude generated by the unadjuvanted vaccine. Thus, the combination of the rapid production of antigens by *E. coli*, combined with a large potency of SBAs, is of great interest in this context.

The humoral and cellular immune responses influence ZIKV pathogenesis and play key roles in controlling ZIKV replication (53). In particular, neutralizing antibodies, induced by ZIKV vaccines, may provide infection avoidance (54), while T cells secrete cytokines to further promote antibody production or directly kill infected cells (55). In the current study, the saponin-based nanoadjuvant enhanced the production of anti-ZIKV neutralizing antibodies, which are important for stimulating protection. In addition, lymphoproliferative responses were observed after *in vitro* stimulation with either the vaccine antigen (zEDIII) or inactivated ZIKV. Using the zEDIII protein as antigen, results show that the IQB80 nanoadjuvant enhanced the immunogenicity of zEDIII, when compared with the unadjuvanted vaccine. In addition, significant anti-ZIKV-specific immune responses were observed in mice immunized with IQB80 nanoadjuvant when compared with alum-adjuvanted mice.

## Concluding Remarks

The results reported here show that immunization with zEDIII together with saponin-based nanoadjuvant IQB-80 from *Q. brasiliensis* is capable of significantly improving immune responses against ZIKV in mice. In addition, the formulation of the nanoadjuvanted vaccine was shown to be safe, with a quick and easily preparation. Moreover, the nanoadjuvant proved to be safe in terms of toxicity. No signs indicative of distress (lethality, local swelling, loss of hair, and piloerection) were observed following the inoculation of any of the preparations evaluated in one or two doses of the immunogens.

Indeed, the nanoadjuvant formulation, especially that containing 10 μg of zEDIII antigen, elicited the production of high IgG antibody titers in relation to the vaccine formulation when adjuvanted with alum. Additionally, this nanoadjuvanted formula was able to induce neutralizing antibodies, as well as antibodies of high avidity. The nanoformulation promoted a positive *in vitro* cell proliferation response to recombinant zEDIII, as well as to an inactivated ZIKV antigen. Moreover, the magnitude of immune responses in alum-adjuvanted mice was lower than in the group that was immunized with the nanoadjuvant. Overall, this study identified saponin-based nanoadjuvants as a suitable adjuvant for ZIKV recombinant vaccines; findings have important implications for subunit vaccines against enveloped viruses, including other flaviviruses. Furthermore, this is a potent adjuvant obtained from renewable sources, which seems to compare favorably with other available alternatives.

## Data Availability Statement

The original contributions presented in the study are included in the article/Supplementary Material, further inquiries can be directed to the corresponding author/s.

## Ethics Statement

All procedures were carried out in accordance with the National Council for Animal Experimentation Control and approved by the institutional Animal Care and Use Committee (CEUA) under protocol UP 5402/17.

## Author Contributions

SC, AV, MC, and FS designed and performed most of the experiments, analyzed data, and wrote the manuscript. TT and MC contributed to some experimental conception and design. SC, AV, TT, PS, MC, and FS analyzed and/or interpreted the data. SC, MC, PR, and FS wrote the manuscript. All authors were involved in critically revising the manuscript for important intellectual content.

## Conflict of Interest

The authors declare that the research was conducted in the absence of any commercial or financial relationships that could be construed as a potential conflict of interest.

## Supplementary Material

Supplementary Figure 1 zEDIII purification using Ni-chelating chromatography. zEDIII recombinant protein produced in *E. coli* BL21 (DE3) strain was solubilized (S) as described in Material and Methods and applied onto Ni-chelating sepharose resin. After washing the resin with 20 mM imidazole (W) protein elution was performed using 0.2 (E1-E2), 0.5 (E3-E5), and 1 M imidazole in lysis buffer.

Supplementary Figure 2 Indirect immunofluorescence against ZIKV 17-infected Vero cells. Vero cells infected with ZIKV 17 (0.01 m.o.i.) were submitted to indirect immune fluorescence assays (IFA) using a pool of sera from naïve mice (background control).

Supplementary Figure 3 Immunoperoxidase monolayer assay (IPMA). Vero cells (in a 96 well plate) were infected with ZIKV 17 (0.01 m.o.i.) and incubated for 4 days at 37°C in a CO_2_ incubator. After incubation, the cell monolayers were fixed in acetone, rehydrated with PBS and incubated with 50 μL of appropriate dilutions of human convalescent serum or a naïve serum for 30 min at 37°C. Anti-immunoglobulin G (anti-IgG)-peroxidase conjugate (Sigma-Aldrich, USA) was used as a secondary antibody. ZIKV-infected cells were revealed by the addition of carbazole (3-amino-9-ethylcarbazole; Sigma-Aldrich, USA). **(A)** Workflow of ZIKV IPMA. **(B)** IPMA using a human convalescent serum, showing ZIKV-infected Vero cells (carmine stained). **(C)** IPMA using a naïve serum (background control).
