# Supplementary figures and images for: Zika Virus Envelope Domain III Recombinant Protein Delivered With Saponin-Based Nanoadjuvant From Quillaja brasiliensis Enhances Anti-Zika Immune Responses, Including Neutralizing Antibodies and Splenocyte Proliferation
Source: Front Immunol. 2021 Mar 4;12:632714. doi: 10.3389/fimmu.2021.632714 (PMC7969523; doi:10.3389/fimmu.2021.632714)

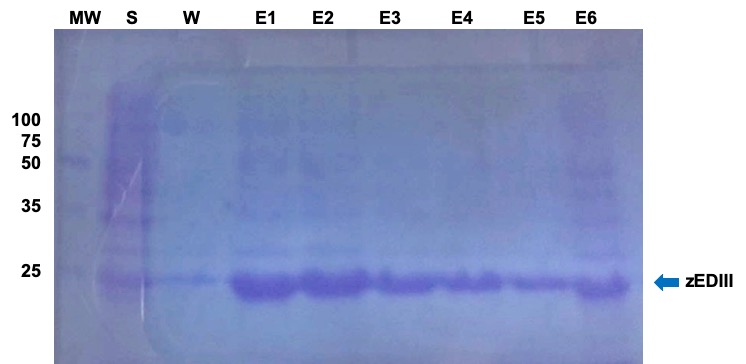

Supplement: Supplementary Figure 1 — zEDIII purification using Ni-chelating chromatography. zEDIII recombinant protein produced in E. coli BL21 (DE3) strain was solubilized (S) as described in Material and Methods and applied onto Ni-chelating sepharose resin. After washing the resin with 20 mM imidazole (W) protein elution was performed using 0.2 (E1-E2), 0.5 (E3-E5), and 1 M imidazole in lysis buffer. [file Image_1.jpeg]

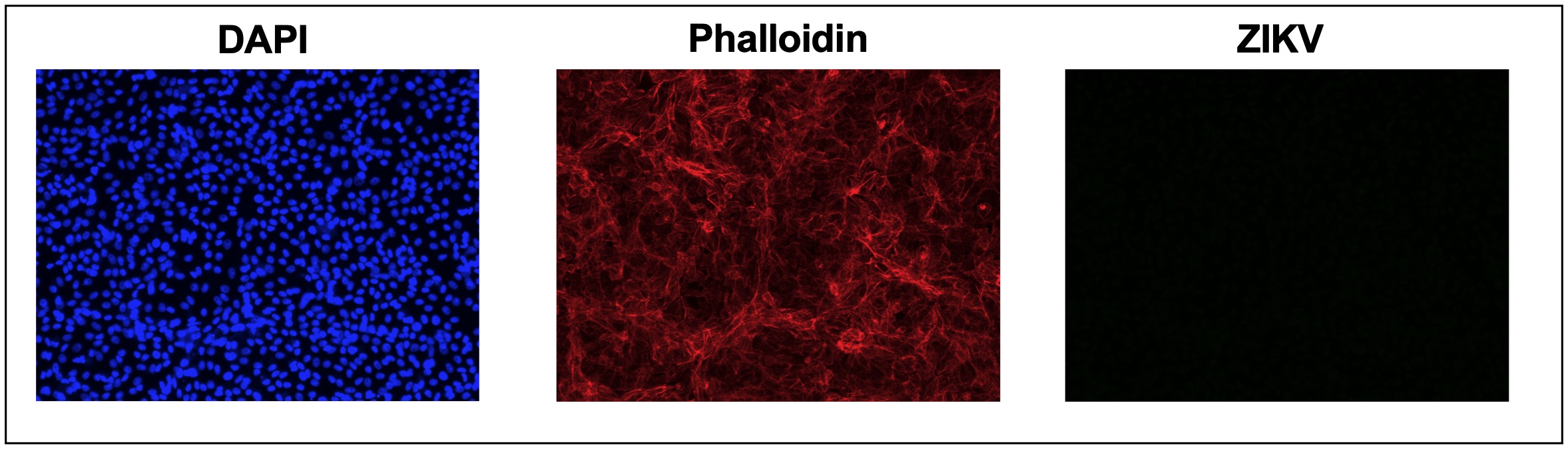

Supplement: Supplementary Figure 2 — Indirect immunofluorescence against ZIKV 17-infected Vero cells. Vero cells infected with ZIKV 17 (0.01 m.o.i.) were submitted to indirect immune fluorescence assays (IFA) using a pool of sera from naïve mice (background control). [file Image_2.jpeg]

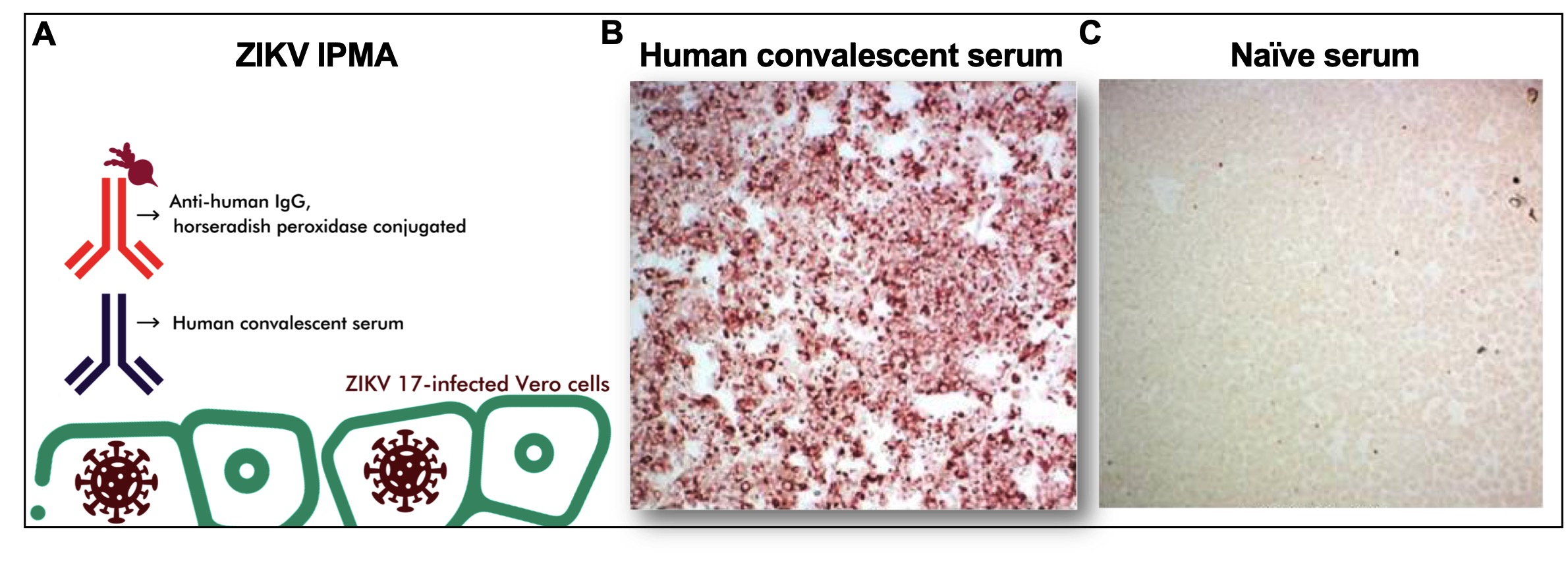

Supplement: Supplementary Figure 3 — Immunoperoxidase monolayer assay (IPMA). Vero cells (in a 96 well plate) were infected with ZIKV 17 (0.01 m.o.i.) and incubated for 4 days at 37°C in a CO2 incubator. After incubation, the cell monolayers were fixed in acetone, rehydrated with PBS and incubated with 50 μL of appropriate dilutions of human convalescent serum or a naïve serum for 30 min at 37°C. Anti-immunoglobulin G (anti-IgG)-peroxidase conjugate (Sigma-Aldrich, USA) was used as a secondary antibody. ZIKV-infected cells were revealed by the addition of carbazole (3-amino-9-ethylcarbazole; Sigma-Aldrich, USA). (A) Workflow of ZIKV IPMA. (B) IPMA using a human convalescent serum, showing ZIKV-infected Vero cells (carmine stained). (C) IPMA using a naïve serum (background control). [file Image_3.jpeg]
